# Osteo-Proliferative Lesions of the Phalanges on Radiography: Associations with Sex, Age, and Osteoarthritis

**DOI:** 10.3390/diagnostics12030618

**Published:** 2022-03-02

**Authors:** Sandra Hermann, Iris Eshed, Iván Sáenz, Niclas Doepner, Katharina Ziegeler, Kay Geert A. Hermann

**Affiliations:** 1 Department of Rheumatology and Clinical Immunology, Charité—Universitätsmedizin Berlin, Corporate Member of Freie Universität Berlin and Humboldt-Universität zu Berlin, 10117 Berlin, Germany; sandra.hermann@charite.de; 2 Sheba Medical Center, Department of Radiology, Sackler Faculty of Medicine, Tel Aviv University, Tel Aviv 6997801, Israel; iriseshed@gmail.com; 3 Departamento de Anatomía, Facultad de Medicina, Universidad de Barcelona, 08007 Barcelona, Spain; dr.ivan.saenz@gmail.com; 4 Department of Radiology, Charité—Universitätsmedizin Berlin, Corporate Member of Freie Universität Berlin and Humboldt-Universität zu Berlin, 10117 Berlin, Germany; niclas.doepner@charite.de (N.D.); katharina.ziegeler@charite.de (K.Z.)

**Keywords:** radiography, peripheral joints, periosteum, arthritis, osteoarthritis

## Abstract

Objectives: The effects of aging such as osteophyte formation, acral shape changes, cortical tunneling, and bone porosity as well as enthesophytes can be studied in the X-rays of hands. However, during the interpretation of radiographs of the hands, misinterpretation and false-positive findings for psoriatic arthritis often occur because periosteal proliferations of the phalanges are overinterpreted and too little is known about enthesophytes of the phalanges in this area. Method: It included a total of 1153 patients (577 men, 576 women) who presented themselves to the emergency department and received a radiography of their right hand to exclude fractures. The Osseographic Scoring System was used in a modified form to record osteophytes and enthesophytes. A linear regression model for periosteal lesions was computed with age, sex, osteophytes, and global diagnosis as covariables. The inter-reader agreement was assessed using ICC (two-way mixed model) on the sum scores of osteophytes and periosteal lesions. Results: Overall, men exhibited more periosteal lesions, demonstrated by a higher mean sum score of 4.14 vs. 3.21 in women (*p* = 0.008). In both sexes, the second and third proximal phalanx were most frequently affected by periosteal lesions, but the frequencies were significantly higher in men. The female sex was negatively associated with an extent of periosteal lesions with a standardized beta of −0.082 (*p* = 0.003), while age and osteophytes were positively associated with betas of 0.347 (*p* < 0.001) and 0.156 (*p* < 0.001), respectively. The distribution of osteophytes per location did not differ between men and women (*p* > 0.05). The inter-reader agreement was excellent for periosteal lesions with ICC of 0.982 (95%CI 0.973–0.989, *p* < 0.001). Conclusions: Special care should be taken not to confuse normal periosteal changes in aging with periosteal apposition in psoriatic arthritis.

## 1. Introduction

The hand is more available than any other body region for detailed analyses of its skeletal elements. The hand was not only the first human object to be X-rayed, but also the foundation of musculoskeletal radiology [1,2]. The effects of aging such as osteophyte formation, acral shape changes, cortical tunneling and bone porosity as well as enthesophytes can be studied. However, a number of inflammatory and metabolic diseases also manifest in the joints and phalanges of the fingers, most notably rheumatoid arthritis (RA) and psoriatic arthritis (PsA), but also hyperparathyroidism. However, while osteo-proliferative changes are uncommon in RA and hyperparathyroidism, they are considered the diagnostic hallmark in PsA, in addition to characteristic erosions [3,4,5]. These osteo-proliferative changes are typically located directly adjacent to the affected joint and are included in the classification criteria for PsA (CASPAR criteria) [6].

As a general rule, osteophytes are sharply demarcated bony prominences that occur at the edge of the articular surfaces. In contrast to this, periosteal attachments in psoriatic arthritis are ill-defined in the early stage, but are also localized at the edge of the joints [7]. Only later in the course of the disease it may smooth out and then look strikingly similar to osteophytes. Periosteal changes in psoriatic arthritis are also described on the shaft of the phalanges as so-called periostitis. These are initially fuzzy calcified lamellae on the diaphyses, which can be transformed into more solid proliferations in the course of the disease. However, enthesophytes also tend to develop in the same location along the shaft during life, which may be due to mechanical stress applied on the hands.

Although computed tomography (CT) has been used in clinical studies to depict osteo-proliferative changes [8], radiography remains the main imaging modality for their detection [9].

Since little is known about enthesophytes of the phalanges, periosteal proliferations of the phalanges are commonly misinterpreted into false-positive findings for psoriatic arthritis.

The Osteographic Score (OSS) is a composite scale for evaluating age-related changes in the skeleton of the hands [10,11]. It uses a combination of osteoporosis and osteoarthritis parameters such as bone porosity, bone proliferation including enthesis ossification, sclerosis, and joint space narrowing [11]. As such, it is well suited as a basis for a modified assessment score in this study, particularly because of its rating of bone proliferation.

In order to increase the body of knowledge regarding osteo-proliferative lesions of the phalanges and to provide a basis for differentiating these changes from psoriatic arthritis, we aimed in the current study to define the frequency, localization, and gender dependence of periosteal attachments of the fingers’ phalanges in the general population and to relate them to degenerative changes of the finger joints.

## 2. Materials and Methods

### 2.1. Patients and Sample Size Estimation

Included in this retrospective cross-sectional study were patients reporting with traumatic pain to the emergency department that underwent a radiography of the right hand to exclude fractures. In order to describe the effects of sex and age on the lesions studied, men and women from different age groups were included in equal proportions. A sample size estimation was calculated by the institute of biometry and clinical epidemiology of our university using a dedicated software (nQuery Version 7.0). Under the assumption of a prevalence of 25% of target lesions in the general population, a sample size of 1153 was held sufficient to measure 95% Confidence interval with a width of 5%. The local ethics committee approved of this study (ID EA2/138/20) prior to data acquisition and individual informed written consent was waived because of the retrospective nature of the investigation. 

### 2.2. Anatomical Studies

**Figure 1:**
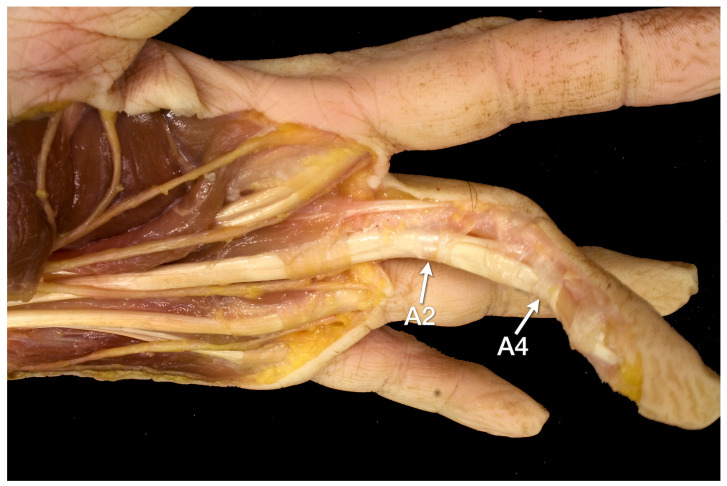
Preparation of the flexor tendons of the 3rd finger and their pulleys with view from oblique palmar. The A2 and A4 annular ligaments (arrows), which insert into the diaphyses of the proximal phalanx and middle phalanx, respectively, can be seen as very delicate structures.

The analysis of the X-ray changes was accompanied by a careful analysis of the underlying anatomy. For this purpose, the cadaver of one finger was dissected in layers. Briefly, after removing the skin from the palmar and incising the palmar aponeurosis, incisions were made along the midline of the fingers as deep as the tendon sheaths. The fatty and connective tissue of the fingers and palm as well as the tendon sheaths were removed while preserving the annular ligaments A1 to A5 and the cruciate ligaments C1 to C3. Special attention was paid to the annular ligaments A2 and A4, according to the research question of this study (Figure 1). 

### 2.3. Imaging and Scoring System

X-ray images of the right hand were assessed in two planes (anterior-posterior and oblique) by one primary reader, a rheumatologist (S.H.) skilled in scoring hands and feet with 12 years of experience. A random sample of 100 patients was also scored to assess inter-reader agreement by a second reader, a radiologist with six years of experience in skeletal imaging (K.Z.). Prior to image scoring, a consensus reading of 20 test patients not included in the analysis was performed by the two readers together with an expert in musculoskeletal imaging (K.G.H.). 

**Figure 2:**
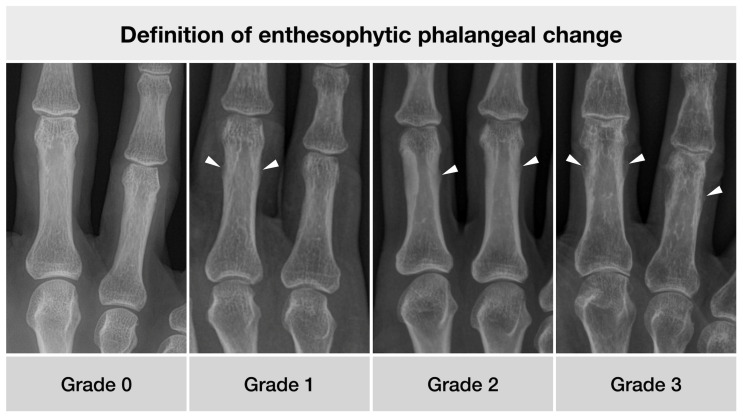
Anterior-posterior X-ray examples of the different grades of enthesophytic growth. Patients’ characteristics from left to right: 53 y/o female, 59 y/o male, 57 y/o female, 82 y/o male.

The Osseographic Score (OSS) [10] was used in a modified form to record the alterations of age in the skeleton with reference to the research question [12]. As the analyses of project presented here did focus on enthesophytic phalangeal change, this domain was further refined. For this purpose, a detailed description of the various grades of enthesophytes (0–3) was formulated (Table 1) and pictorially represented in an atlas (Figure 2).

**Figure 3:**
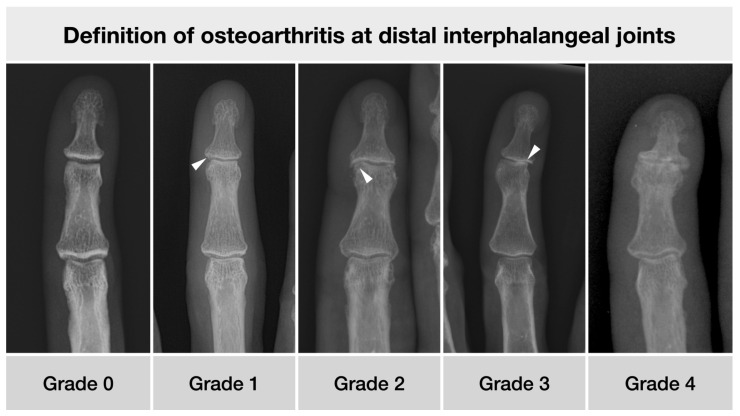
The different degrees of osteoarthritis formation at the distal interphalangeal joints. 0—normal shape; 1—minimal de-rounding (arrowhead) and minute cystic changes; 2—gross osteophytes and cyst formation (arrowhead), joint space yet well preserved; 3—marked asymmetric joint space narrowing (arrowhead); 4—complete joint space narrowing and marked osteophyte formation. Patients’ characteristics from left to right: 46 y/o male, 59 y/o female, 62 y/o male, 63 y/o female, 69 y/o female.

In each subject, the proximal, middle, and distal phalanges were scored for the presence of enthesophytes. A sum score was then calculated for each subject. The osteoarthritis of the metacarpophalangeal and interphalangeal joints was also recorded using the Kellgren and Lawrence score adapted for small joints [13], with a scale from 0 (absent) to 4 (severe). Examples are given in Figure 3. In addition, a global imaging diagnosis was made by the expert reader, distinguishing between degenerative, inflammatory, or neither. The degenerative category included all cases with evidence of either osteophytes, joint space narrowing, sclerosis, or a combination thereof on the hand at any joint. Inflammatory status was assigned if erosions of the joints were apparent, again at any joints of the hand. Single joints and entheseal sites in a fractured bone were excluded. 

### 2.4. Statistical Analysis

All statistical analyses were performed using SPSS version 27 with a significance level of *p* < 0.05. All analyses were primarily performed for men and women separately, and in the case of sex, differences were also reported separately. Frequencies of lesions per region were compared using Chi^2^ tests. Spearman’s rho was computed to assess the correlation of age with periosteal lesions and OA. A linear regression model for periosteal lesions was computed with age, sex, OA (sum score), and radiographic diagnosis as covariables. The inter-reader agreement was assessed using ICC (two-way mixed model) on the sum scores of OA and periosteal lesions. 

## 3. Results

### 3.1. Patients 

As determined by the sample size estimation, 1153 patients were included in this retrospective analysis, with equal proportions of men (*n* = 577) and women (*n* = 576). A global imaging diagnosis of degenerative disease was assigned in 352 (30.5%) and inflammatory disease was seen in 59 (5.1%) of the patients. In 152 patients, one or more locations were excluded from the analysis, due to a fracture.

### 3.2. Osteoarthritis

The distribution of osteoarthritis per location did not differ between men and women (*p* > 0.05). A complete table of frequencies of OA (Kellgren and Lawrence Grade 2 or higher) at all locations and for different age groups is given as Table 2. In all fingers, the DIP joints were most frequently affected, followed by the PIP joints. The second and third fingers were more affected than the fourth and fifth; as expected, OA was much more prevalent in older patients. Correlation analyses showed a strong association of extent of OA (expressed as sum scores) and patient’s age with a Spearman’s rho of 0.660 (*p* < 0.001).

### 3.3. Periosteal Lesions

**Figure 4:**
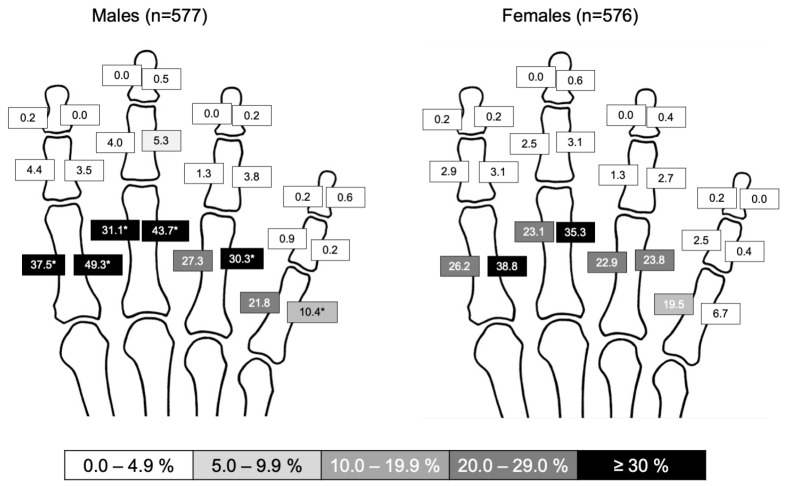
Distribution of periosteal lesions at phalanges. Relative frequencies (%) per region. Significantly higher frequencies are marked with an asterisk (*); *p*-values derived from Chi^2^ tests.

Overall, men exhibited more periosteal lesions, with a higher mean sum score of 4.14 vs. 3.21 in women (*p* = 0.008). A graphical illustration of lesions per region is given as Figure 4. In both men and women, the second and third proximal phalanx were most frequently affected by periosteal lesions, but frequencies were significantly higher in men.

In both men and women, the extent of periosteal lesions correlated with age and OA with Spearman’s rho of 0.586 (*p* < 0.001) and 0.535 (*p* < 0.001) in men and 0.575 (*p* < 0.001) and 0.562 (*p* < 0.001) in women. The regression analysis yielded a corrected R^2^ of 0.265 for the model including sex, age, sum score for OA, and global category (none/degenerative/inflammatory) as covariables. The female sex was negatively associated with an extent of periosteal lesions with a standardized beta of −0.082 (*p* = 0.003), while age, extent of OA and the degenerative/inflammatory category were positively associated with betas of 0.347 (*p* < 0.001), 0.156 (*p* < 0.001), and 0.078 (*p* = 0.041), respectively. 

### 3.4. Inter-Reader Agreement

The inter-reader agreement was excellent for both periosteal lesions and osteoarthritis with ICCs of 0.982 (95%CI 0.973–0.989, *p* < 0.001) and 0.989 (95%CI 0.984–0.993, *p* < 0.001), respectively.

## 4. Discussion

This study adds to the body of knowledge on the natural history of skeletal aging of the hand, demonstrating osteophyte and periosteal enthesophytes of the phalanges in a central-European patient population. We found a high frequency of osteo-proliferative lesions at the proximal phalanx of the second and third finger. Furthermore, the extent of these lesions correlated with both the age and grade of the osteoarthritis of the finger joints.

Our investigation revealed that males exhibited osteo-proliferative lesions more frequently than females, while the distribution of finger OA did not differ between the sexes. These findings are in line with those of Kalichman et al., who studied the association of midshaft enthesophytes and osteophytes and found that age corresponded to 45% of enthesophyte variation in males but only 25% in females [14]. This finding may result from a stronger grip of males compared to females [15] resulting in a more developed pulley system at the fingers and also possibly higher mechanic strain on their insertions.

### 4.1. Anatomical Considerations

Given the unclear data on enthesophytes of the fingers, we preceded the radiographic examinations with a precise anatomical dissection. It was found that there are normally no roughnesses or calcareous protrusions on the lateral and medial sides of the proximal and middle phalanges. Periosteal appositions were particularly frequent at the attachments of the A2 annular ligament and the C1 cruciate ligament (frequency in females 6.7–38.8% and in males10.4–49.3%) at the basal phalanges. Periosteal lesions were rare at the middle and distal phalanges. Anatomically, the A2 and A4 annular ligaments are of particular importance, as they prevent the superficial and deep flexor tendons from snapping out of their sheaths during finger flexion. The lumbrical muscles play no role in the development of phalangeal enthesophytes because both their origin and insertion are on the flexor and extensor tendons, respectively [16], and thus have no bony contact. Periosteal lesions were rare at the middle and distal phalanges. The work of Meng et al. deals with a similar aspect, namely the palmar ridges of the phalanges [17]. These are particularly visible on oblique images of the fingers, in contrast to the lesions in the focus of our study, which are clearly visible on anterior-posterior projections. The histological analyses by Meng and colleagues show a fibrocartilaginous layer between the attachments of the annular ligaments and the palmar edges of the phalanges, which clearly identifies these regions as entheses [17,18].

### 4.2. Psoriatic Arthritis

Periosteal changes on the hands are the typical sign of psoriatic arthritis and some forms of peripheral spondyloarthritis [19]. These are known to be spiculated in appearance and often localized to the acral and articular sites. However, it has also been postulated that periosteal proliferations occur diaphyseally in the setting of periostitis. However, their frequency on radiographs has not been well studied. To complicate matters, diaphyseal periosteal attachments also occur in the form of physiologic enthesophytes, leading to diagnostic uncertainty. Our data underlines the limited specificity of diaphyseal periosteal attachments, especially in the proximal phalanges and in men more than in women. In patients with suspected psoriatic arthritis, changes in these localizations should be interpreted with care. Conversely, there is also a diagnostic dilemma when patients with the clear clinical condition of psoriatic arthritis are presented as normal in the radiological report, either because the periosteal changes have been inadequately analyzed or the early-stage disease does not yet make these visible on X-rays.

### 4.3. Limitations

The study was planned with a long lead time, but some limitations need to be discussed. The X-ray examinations of the right hand from the emergency room were selected for the analysis on the assumption that the acute traumatic changes, if any, do not cause dependence on chronic ossification of the cruciate and annular ligament structures of the fingers. Likewise, conclusions about mechanical loads should be drawn with caution because, due to the nature of this study, we were unable to collect data on previously known hand osteoarthritis or on occupational or recreational loads on the finger joints. Purely theoretically, however, there is a risk of confusion of an acute osseous avulsion of the annular ligament avulsion with a chronic enthesophyte. However, this influence can be discarded as relevant due to the rarity of osseous avulsions at the phalanges.

## 5. Conclusions

Enthesophytes that can be reliably detected by conventional radiography represent a form of ageing and correlate with the degree of osteoarthritis of the finger joints. Due to the significantly lower manifestation in the female sex, mechanical causes can be postulated as the cause of the changes. In the diagnostic process, the physiological periosteal changes should not be confused with those of diseases such as hyperparathyroidism or psoriatic arthritis. In our future projects, we will target patients with known psoriatic arthritis and also analyze the association of entheseal bone proliferations with laboratory biomarkers of bone metabolism.

## Figures and Tables

**Table 1:** Refined definition of enthesophytic growth at digital phalanges, modified from Karasik et al. [12].

Grade	Definition of Enthesophytic Phalangeal Change
0	Cortical bone smooth without prominences
1	Irregular cortical bone, possibly with fluffing, possibly with smallest, flattened periosteal proliferations
2	Cortical bone with well-defined enthesophytic protuberance, not more than 1 mm of substance increase
3	Cortical bone with clearly recognizable enthesophytic proliferation of 1 mm or more

**Table 2:** Frequency of OA. Frequencies given as relative (%) and absolute numbers.

	<30 Years (%, n)	30–39 Years (%, n)	40–49 Years (%, n)	50–59 Years (%, n)	60–69 Years (%, n)	70–79 Years (%, n)	≥80 Years (%, n)
**DIP-II**	2.0 (5/252)	2.4 (4/168)	10.6 (18/170)	16.4 (26/159)	34.1 (60/176)	50.3 (75/149)	49.4 (39/79)
**PIP-II**	0.0 (0/252)	1.2 (2/168)	0.0 (0/170)	4.4 (7/159)	7.4 (13/176)	16.8 (25/149)	21.5 (17/79)
**MCP-II**	0.0 (0/252)	0.0 (0/168)	0.6 (1/170)	2.5 (4/159)	2.8 (5/176)	4.7 (7/149)	12.7 (10/79)
**DIP-III**	1.6 (4/252)	4.3 (7/168)	5.9 (10/170)	16.4 (26/159)	31.8 (56/176)	55.7 (83/149)	48.1 (38/79)
**PIP-III**	0.4 (1/252)	0.0 (0/168)	1.8 (3/170)	3.8 (6/159)	6.3 (11/176)	14.8 (22/149)	20.3 (16/79)
**MCP-III**	0.0 (0/252)	0.0 (0/168)	1.2 (2/170)	1.9 (3/159)	2.3 (4/176)	6.7 (10/149)	13.9 (11/79)
**DIP-IV**	0.4 (1/252)	4.2 (7/168)	6.5 (11/170)	13.2 (21/159)	22.7 (40/176)	39.6 (59/149)	36.7 (29/79)
**PIP-IV**	0.0 (0/252)	1.2 (2/168)	2.9 (5/170)	5.7 (9/159)	7.4 (13/176)	22.8 (34/149)	22.8 (18/79)
**MCP-IV**	0.0 (0/252)	0.0 (0/168)	0.0 (0/170)	0.0 (0/159)	0.0 (0/176)	2.7 (4/149)	1.3 (1/79)
**DIP-V**	0.4 (1/252)	3.0 (5/168)	5.9 (10/170)	8.2 (13/159)	21.0 (37/176)	34.2 (51/149)	43.0 (34/79)
**PIP-V**	0.0 (0/252)	0.6 (1/168)	0.0 (0/170)	3.1 (5/159)	10.8 (19/176)	20.1 (30/149)	24.1 (19/79)
**MCP-V**	0.0 (0/252)	0.0 (0/168)	0.0 (0/170)	0.0 (0/159)	0.6 (1/176)	1.3 (2/149)	3.8 (3/79)

## Data Availability

The data presented in this study are available on request from the corresponding author.
